# Noninvasive Fuel Flow Monitoring System for Vented Fuel Oil Heaters

**DOI:** 10.3390/s23125664

**Published:** 2023-06-17

**Authors:** Baxter Bond, Alana Vilagi, Dominique J. Pride

**Affiliations:** Alaska Center for Energy and Power, University of Alaska Fairbanks, Fairbanks, AK 99775, USA; atvilagi@alaska.edu (A.V.); djpride@alaska.edu (D.J.P.)

**Keywords:** flow monitoring, home energy monitoring, magnetoresistive devices, Arduino, residential heating

## Abstract

In this work, we present hardware and firmware design and preliminary testing results for a noninvasive device for measuring fuel oil consumption in fuel oil vented heaters. Fuel oil vented heaters are a popular space heating method in northern climates. Monitoring fuel consumption is useful to understanding residential daily and seasonal heating patterns and understanding the thermal characteristics of buildings. The device is a pump monitoring apparatus (PuMA) that employs a magnetoresistive sensor to monitor the activity of solenoid driven positive displacement pumps, which are commonly used in fuel oil vented heaters. PuMA accuracy for calculating fuel oil consumption was evaluated in a lab setting and found to vary up to 7% from the measured consumption value during testing. This variance will be explored more in field testing.

## 1. Introduction

It is estimated that space heating accounts for more than 70% of residential energy consumption in Alaska [1]. Coupled with the high heat load is the high cost of resources, with heating fuel oil reaching more than $10 per gallon in some remote Alaskan villages [2]. Many homes in Alaska use fuel oil as a main or supplemental source of heating, owing to its ease of transport, minimal infrastructure requirements compared to natural gas, and a multitude of well-developed oil-fired heating system options, including furnaces and boilers.

To mitigate these costs, organizations such as the Alaska Housing Finance Corporation and the Alaska Energy Authority work with communities to provide energy efficiency and weatherization programs [3,4,5]. Because heating fuel is often the primary source of heat in rural communities, heat loads can be calculated by measuring fuel oil consumption. However, the lack of consistent and accurate heating fuel data means it is difficult to measure the impact on fuel consumption of any energy efficiency measure implemented and to quantify the associated cost savings [5]. Having a simple way to gather data on heating fuel consumption and thus heat loads would provide baseline data useful for housing and energy programs across the state.

There are primarily two approaches for predicting building energy consumption: physical modeling that relies on thermodynamic rules with inputs of detailed building and environmental parameters and data-driven modeling that relies on archived data [6]. Physical modeling is limited by the accuracy of its inputs. It is difficult to estimate residential heating loads due to humidity, temperature fluctuations, wind exposure, solar gain, occupancy, and occupant behavior [7]. Data-driven modeling relies on the availability of measured data, which is limited for Alaskan residential buildings [5].

A common indoor heating system used throughout Alaska is the fuel oil vented heater. Colloquially known as “Toyostoves”, the most popular heaters of this style are manufactured by Toyotomi. The Monitor brand fuel oil vented heater is still in use throughout many Alaskan residences, but the manufacturer, Hitachi, ceased parts production in 2014 [8]. Fuel oil vented heaters intake outside air, combust, transfer heat to the indoors via a heat exchanger and fan assembly, then exhaust the combustion products to the outside. Vented fuel oil heaters are efficient, with those currently on the market claiming 87% efficiency [9]. Compared to furnaces and boilers, vented fuel oil heaters are often the preferred form of heating system, owing to their relatively low cost, simple installation, and ease of operation.

These heaters regulate room air temperature with a thermistor string and transfer fuel oil via a positive displacement solenoid pump that is controlled by the main motherboard [10,11]. The pump is controlled by a 150–160 volts of direct current (Vdc) pulse lasting 4.4 milliseconds per cycle. The rate of fuel flow is determined by the frequency at which the pulses are sent; the width of the pulse remains constant. The fuel flow rate ranges from 0.151 liters per hour (lph), rated, on the lowest setting of the smallest heater model, to 1.139 lph, rated, on the highest setting of the largest heater model.

Currently, there are limitations to monitoring heating fuel oil consumption noninvasively (i.e., without breaking into the fuel line). Although in-line flow meters have a higher accuracy, they are often expensive and inconvenient to install [12]. The Cold Climate Housing Research Center evaluated eight fuel use measurement systems for a Toyostove heater, and found that the fuel flow rate was too low for several of the systems to register the amount of fuel used, and error for run-time fuel monitoring systems was between 13% to 27% [12]. There are tank-level monitoring systems available that monitor fuel levels ultrasonically but are not suited to low outside temperatures experienced in interior Alaskan winters [13,14].

Because of the widespread usage of direct vented fuel oil heaters, we developed a pump monitoring apparatus (PuMA) that noninvasively monitors heating fuel oil consumption by taking advantage of the inherent magnetic field produced by the positive displacement solenoid inside these types of heaters. Using a magnetoresistive (MR) sensor, the presence of a magnetic field can be used as a proxy for fuel flow as this field is what drives the piston to pump heating fuel oil into the burner pot. Counting the number of times the presence of a magnetic field is detected is equal to the number of times the piston is cycling, moving a measurable volume of heating fuel oil each cycle. Monitoring the activity of the positive displacement solenoid can be used in calculating heating fuel oil consumption.

## 2. Basic Concept

### 2.1. Pump Frequency

The frequency of the fuel pump was measured with a portable digital oscilloscope with a 10X attenuator probe clipped directly to the leads on the solenoid pump. The frequencies of the combustion settings for a Toyostove Laser 56/560, the fuel oil vented heater used in the experimental protocol, are displayed in Table 1.

### 2.2. Determine Period

Period is the inverse of frequency. The periods for a Toyostove Laser 56/560 are displayed in Table 2.

### 2.3. Volume per Pump Cycle Equation

Flow rates are from the Toyostove service manual [10] and displayed in Table 3.

### 2.4. Fuel Displacement

The amount of heating fuel volume displaced per pump cycle (L/cycle) is calculated by the following equation:

(1) $$liters/cycle=flowrate\left(\frac{l}{hr}\right)*\left(\frac{hr}{3600s}\right)*period^{-1}\left(\frac{1}{s}\right)$$

The approximate fuel displacement based on manual flow rate values for a Toyostove Laser 56/560 pump is displayed in Table 4. However, we found that the amount of fuel displaced with pump cycle varies depending on the fuel type used and the combustion setting in which the heater is operating. These values were found through testing three different common heating fuel oils, and are displayed in Table 5.

## 3. Pump Monitoring Apparatus

### 3.1. Hardware

**Figure 1:**
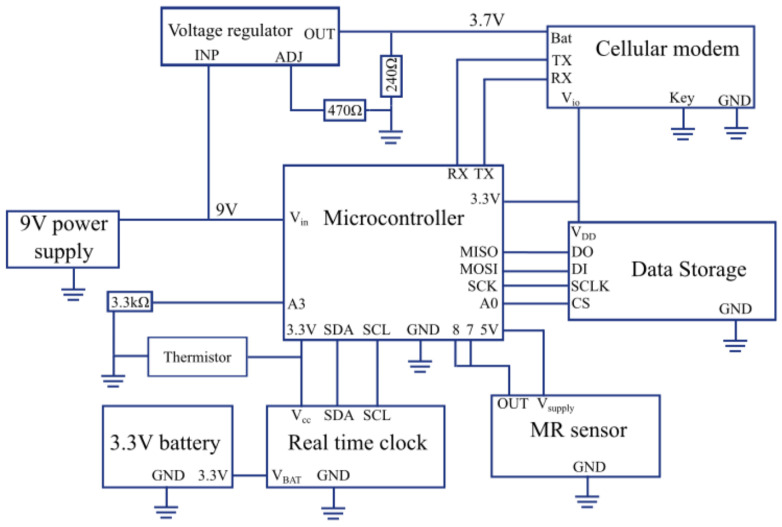
Functional Block diagram.

**Figure 2:**
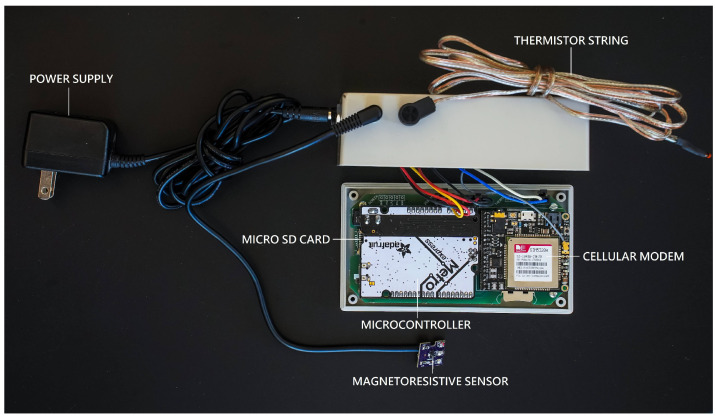
PuMA open with labeled components.

The PuMA is designed to mount on the side of the fuel oil vented heater near access to the solenoid pump. The PuMA is made up of six systems: microcontroller, sensing, storage, transmission, supportive hardware and circuitry, and power supply (Figure 1). The microcontroller, storage, transmission, and supportive hardware and circuitry are contained within a protective casing (Figure 2). The unit is attached to the side of the heater with industrial-strength velcro or similar adhesive.

#### 3.1.1. Microcontroller

The microcontroller utilized is the Adafruit Metro M0 Express [16]. It is based on the 32-bit Atmel SAMD21. The Metro is an ARM microcontroller that consumes low power and uses several communication protocols such as UART, I2C, and SPI. The onboard 10-bit ADC is also used and it maps 0 V to 3.3 V in 1024 steps, where the maximum 3.3 V corresponds to a digital value of 1023. The steps are linear. The clock speed is 48 MHz.

#### 3.1.2. Sensing

There are two main sensors, a thermistor string and a magnetoresistor. 3.3 Vdc is supplied from the microcontroller to the thermistor string and a resistor. The voltage drop across the resistor is measured using an analog pin at the junction of the thermistor and resistor. The microcontroller measures this drop using its onboard analog to digital converter. Resistance can be calculated from this value. In practice, PuMA’s thermistor is placed next to the heater’s thermistor string to monitor the same temperature as the heater. Temperatures tend to vary significantly, e.g., next to a window compared with next to a wall, so the behavior of the heater will change depending on the placement of its thermistor.

The magnetoresistive sensor attaches to the solenoid pump with a clip or similar mechanism [17]. It uses the 2SS52M magnetoresistor in the S552MT package. It is supplied with 5 Vdc coming from the microcontroller. The output of the magnetoresistor is stepped down to 3.3 Vdc using a voltage divider. It is open drain; when a magnetic field is detected, the output goes to 0 V. The sensor is roughly 15 mm by 15 mm, which is small enough to easily fit on the exposed side of a solenoid pump.

#### 3.1.3. Storage

Since the microcontroller does not have any inherent data storage capabilities, an 8 GB Micro SD card manufactured by SanDisk was incorporated. The communication protocol used was SPI. Average operation uses approximately 6 kB of storage per day.

#### 3.1.4. Transmission

An Adafruit FONA 3G is used for nonlocal storage by sending data via a cellular network to an FTP server or a third party Internet of Things (IoT) service [18]. Wireless long-distance communication allows for nonlocal processing of data.

#### 3.1.5. RTC and Passive Components

A DS3231 temperature-compensated real-time clock is used for timekeeping [19]. Timestamps are necessary to calculate fuel oil consumption over time. If there is a power interruption, the RTC automatically switches to a CR1632 battery.

The rest of the components are passive, namely, resistors and capacitors. These components are necessary for the operation of the device, such as pullup resistors, voltage dividers, and decoupling capacitors.

#### 3.1.6. Power Supply

The unit is powered by a 9 Vdc 1 A switching AC to DC power adapter. The microcontroller is directly powered by the 9 Vdc power supply. An LM317 adjustable voltage regulator powers the FONA at approximately 3.7 V [20].

### 3.2. Firmware

#### 3.2.1. Microcontroller Setup

Once the microcontroller receives power, it enters the setup routines with the following steps:

1. Initialize the SD card
2. Read the configuration files from the SD card and implement defined variables
3. Initialize UART serial communication with the FONA
4. Update the FONA clock using HTTP time protocol if there is network signal
5. Clear the I2C bus in case the RTC has frozen, and begin communication with the RTC
6. Sync the RTC with the updated FONA time
7. Create file directories if nonexistent
8. Initialize the magnetoresistor to both a digital input and an interrupt pin, and the thermistor to an analog input pin
9. Load any persistence files saved from the last time the PuMA was powered.

**Figure 3:**
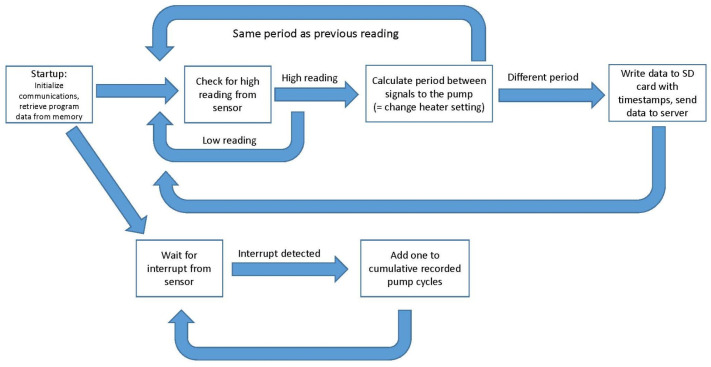
PuMA software routine.

During the whole setup process, the PuMA also prints to the USB serial output, providing feedback from the PuMA during installation and allowing troubleshooting of any anomalies in the setup period. From there, it enters its main loop (Figure 3).

#### 3.2.2. Main Loop

Interrupt: Through the duration of the main loop, there is an interrupt pin that detects when the magnetoresistor senses a field and therefore fuel pump activity. When the interrupt is activated, it quickly adds one to the cumulative count of the individual cycles.

Find period: In the main loop, the PuMA looks for input from the magnetoresistor, which indicates pump activity. If there is no activity for more than 1 s, the heater is considered off. If there is activity, the PuMA will measure the difference in time between four points to get three periods. The average is taken of these three periods. The difference is then taken between the average period and the individual period. If there is a difference of more than 10 ms, it restarts gathering 3 periods. If not, it uses the average period to proceed through the program.

Determine mode from period: The period is then compared to a range of values calculated from the configuration file. Depending on what range the period falls into, it is characterized into high, medium, or low mode. These correspond to the heating modes of a standard Toyotomi heater. Monitor heaters have four running modes: high, high-medium, low-medium, and low.

Record event-based data: For fuel oil monitoring, the PuMA uses event-based data gathering. If the heater’s mode changes from one 3-period interval to another, it stores the following data to the SD card: Unix timestamp, heater mode, cumulative pump cycles, and thermistor resistance.

#### 3.2.3. Thermistor Resistance

The resistance as measured by the thermistor is calculated by the following equation:

(2) $$R_{therm}=3,375,900/input-3300$$

This equation is derived from the voltage divider equation $V_{out}=V_{in}\times R_{2}/\left(R_{therm}+R_{2}\right)$. $R_{2}$ is equal to 3300 ohms, $V_{in}$ is 3.3 volts. $V_{out}$ correlates with the microcontroller’s analog to digital converter, and its value is between 0 and 1023. 0 corresponds to 0 V, and 1023 means saturation at 3.3 V. Resistance is used to calculate temperature in the data processing stage.

#### 3.2.4. FONA (Optional)

For nonlocal data gathering, the FONA is optionally used. It can send data packets as needed to a third-party cloud service as an IoT device or send data to an FTP server every few hours. The ability to send data heavily depends on the quality and strength of the cellular network in the area.

## 4. Testing

**Figure 4:**
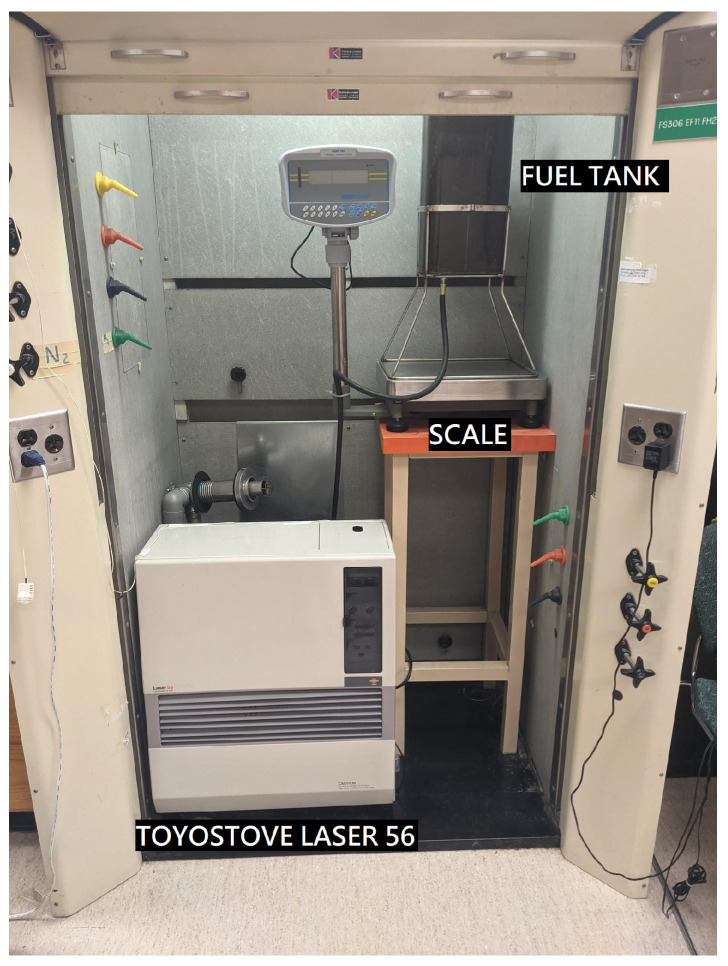
Testing Setup with Toyostove Laser 56 and Adam Equipment GBK scale.

**Figure 5:**
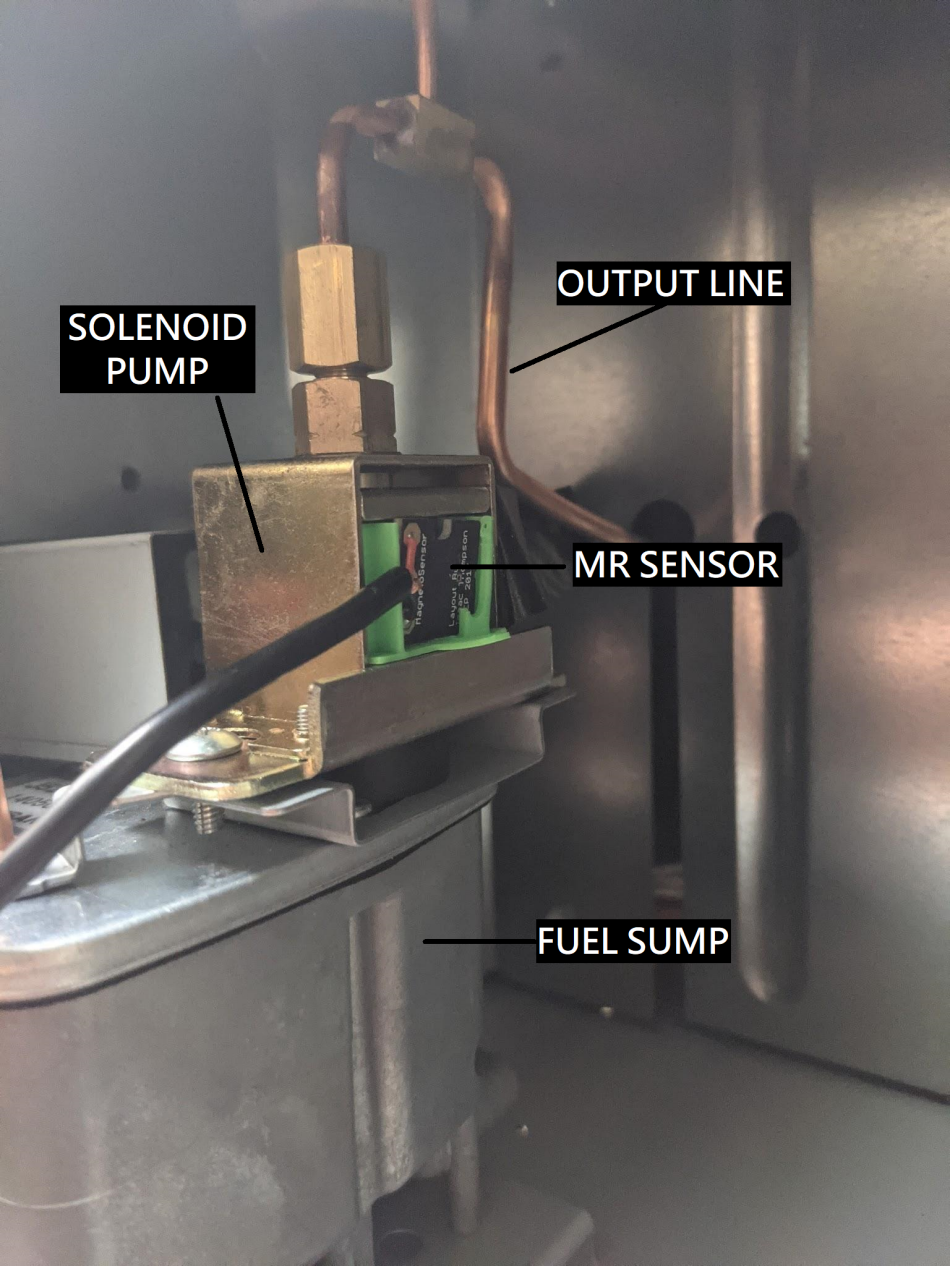
PuMA MR sensor placement on solenoid pump.

The accuracy of the PuMA device was evaluated in a laboratory by monitoring fuel consumption via a data logging scale while simultaneously collecting solenoid pump activity via the PuMA MR sensor. Actual fuel consumed by weight is compared to the calculated amount from the PuMA data and predetermined values for volume displaced per pump cycle. A Laser 56 Toyostove was installed in a fume hood, and an external fuel tank was placed on an ADAM GBK 16a bench scale (Figure 4). The scale records weight at a frequency of one reading per second. The PuMA sensor clip attaches to the outside of the fuel pump (Figure 5), and the PuMA is plugged into an outlet outside of the fume hood. Combustion was automatically operated by the heater circuit in response to indoor temperature readings with the heater’s own thermostat.

Each PuMA has an MR sensor that is placed on the outside of the fuel pump to monitor the solenoid. A clip was designed to hold the MR sensor and continues to be under development. The PuMA is an event-driven device, only logging data when it detects a magnetic field generated by fuel pump activity. The PuMA determines combustion setting of low, medium, or high by keeping a count of the number of times the fuel pump cycles and comparing this to a corresponding range of periods for each setting (see Table 2). Currently, the program is adjusted for the make and model of the heater. The heater operation, electrical pulses, and thermistor resistance are collected and marked with a Unix timestamp. From this data values for fuel consumption, indoor temperature, and flow rate can be calculated. Data recording is event-driven, and the flow rate is considered constant until a change in period is recorded.

## 5. Results

Three Toyostove Laser 56 solenoid pumps of various unknown ages were tested using the procedure described in the Testing section. The amount of fuel consumed as measured by the change in weight of the fuel tank is compared to the calculation using the pump cycles counted by the PuMA and our earlier derived value of the average amount of fuel displaced per pump cycle. Fuel consumption totals were calculated using both the average fuel displaced per pump cycle, and using the individual fuel displacement as associated with the heater’s combustion setting (Table 5).

**Figure 6:**
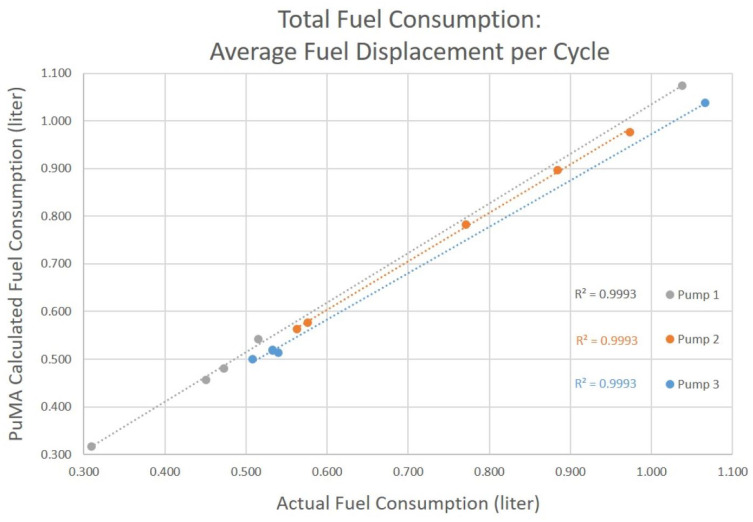
Fuel consumption as calculated by the PuMA, using average fuel displacement per cycle, compared with actual consumption.

Using the average fuel displacement per cycle, the PuMA calculated fuel consumption total varied from −4.8% to +5.3% from the actual value as measured by the scale (Figure 6).

**Figure 7:**
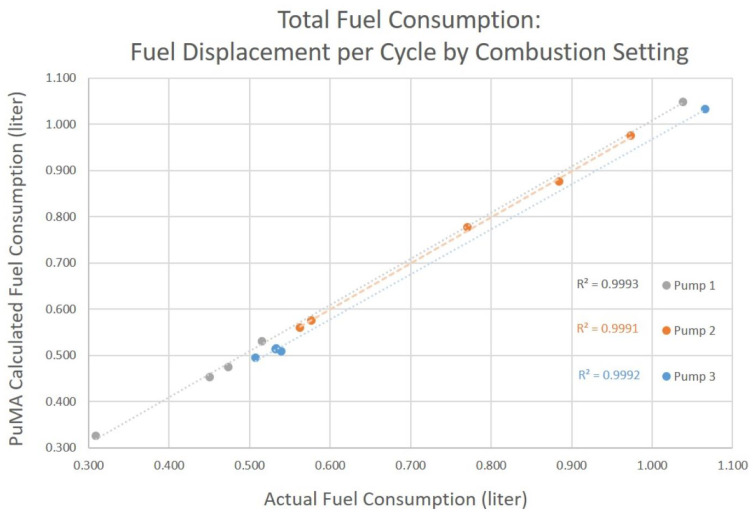
Fuel consumption as calculated by the PuMA, using fuel displacement per cycle by combustion setting, compared with actual consumption.

Using the fuel displacement per cycle by combustion setting, the PuMA calculated fuel consumption total varied from −3.3% to +6.7% from the actual value as measured by the scale (Figure 7).

It was questioned whether a single correction factor (associated with heater model) could be applied to the PuMA calculated fuel consumption to get a better estimate to the actual value. Ideally, all solenoid pumps designed for a particular model of fuel oil vented heater would consistently displace the same amount of fuel with every pump cycle. However, the age of the fuel pump components (e.g., the spring and valve), the type of heating fuel used (e.g., kerosene or heating fuel no. 1), and the setting in which the heater is operating (e.g., high, medium or low) will have an effect on how much fuel is displaced by the pump piston up to 4% [21]. A one-way analysis of variance (ANOVA) was conducted to determine if a statistically significant difference between means existed between the three solenoid pumps that were tested. The null hypothesis assumes that there is no difference between the Laser 56 pump performance; thus, a single correction factor can be applied to obtain a closer approximation to actual fuel consumption. However, it was determined there was a statistically significant difference between groups as determined by one-way ANOVA (F(2,12) = [27.81948], *p* = 3.12 $\times \;10^{-5}$). The results of the ANOVA show the *p*-value is much smaller than 0.05; therefore, a single correction factor for all pumps of a model cannot be applied.

## 6. Discussion

Fuel consumption calculated using the PuMA varied within ±7% from the actual value measured using the scale in lab trials, using both average fuel displacement and that of individual combustion settings in calculations. A one-way ANOVA found that correction factors would need to be determined for each unique heater to obtain a better estimate of fuel consumption. Correction factors will be determined in future explorations of field studies with the PuMA. A broader set of fuel oil vented heaters is needed to expand our understanding of PuMA accuracy to evaluate fuel consumption.

Age of the fuel pump, environmental conditions, and the type of heating fuel used will contribute to the amount of error between actual heating fuel consumption and the amount calculated by the PuMA. Larger studies and longer testing on residential fuel oil vented heaters will lead to a better understanding of this variance.

In instances where the home is only heating with fuel oil, using the PuMA could provide a representation of the building’s heat load by measuring fuel oil consumption. In lab settings, the PuMA performed better than comparable systems available on the market [12].

The PuMA may be a useful tool to organizations trying to evaluate the effectiveness of weatherization programs as well as to individual consumers. Having an understanding of heating usage patterns at the level of individual homes can help consumers lower costs via behavioral changes and efficiency measures. Some factors that consumers have control over when it comes to regulating heating costs include the temperatures users choose to set as their baseline level of comfort and the regularity of their heating periods. Having a tool to inform individuals of their daily heating fuel consumption could lead to long-term cost-saving habits.

## Figures and Tables

**Table 1:** Toyostove Laser 56/560 frequencies.

Frequency (Hz)		
**Low**	**Medium**	**High**
5.70	10.6	16.3

**Table 2:** Toyostove Laser 56/560 periods.

Period (ms)		
**Low**	**Medium**	**High**
66.6	97	166

**Table 3:** Toyostove Laser 56/560 flow rates.

Flow Rate (L/h)		
**Low**	**Medium**	**High**
0.227	0.428	0.625

**Table 4:** Toyostove Laser 56/560 fuel displacement.

Approx. Vol. per Cycle L/Cycle)
1.155 $\times \;10^{-5}$

**Table 5:** Toyostove Laser 56/560 measured Vol. per Cycle (L/cycle).

Measured Vol. per Cycle (L/Cycle)					
**Heating Fluid**	**Density [15]**	**Low**	**Medium**	**High**	**Average**
Kerosene	0.780	1.19 $\times \;10^{-5}$	1.16 $\times \;10^{-5}$	1.10 $\times \;10^{-5}$	1.15 $\times \;10^{-5}$
Heating Fuel No. 1	0.801	1.14 $\times \;10^{-5}$	1.11 $\times \;10^{-5}$	1.08 $\times \;10^{-5}$	1.11 $\times \;10^{-5}$
Heating Fuel No. 2	0.870	1.14 $\times \;10^{-5}$	1.11 $\times \;10^{-5}$	1.06 $\times \;10^{-5}$	1.10 $\times \;10^{-5}$

## Data Availability

Data and certain code is available on request due to privacy restrictions. The data and certain code presented in this study are available on request from the corresponding author. The data/code are not publicly available due to private information in the firmware in order to send data to internal services.

## Abbreviations

The following abbreviations are used in this manuscript:

Vdc	Volts of direct current
lph	liters per hour
PuMA	Pump Monitoring Apparatus
MR	Magnetoresistor
Hz	Hertz
hr	hour
s	seconds
UART	Universal Asynchronous Receiver Transmitter
I2C	Inter-Integrated Circuit
SPI	Serial Peripheral Interface
ADC	Analog-to-Digital Converter
V	volts
MHz	Megahertz
mm	millimeter
GB	gigabyte
SD	secure digital
kB	kilobyte
FTP	file transfer protocol
IoT	Internet of things
RTC	real time clock
AC	alternating current
DC	direct current
HTTP	hypertext transfer protocol
USB	universal serial bus
ms	milliseconds
ANOVA	analysis of variance
